# PrEP Interest Among Men Who Have Sex with Men in the Netherlands: Covariates and Differences Across Samples

**DOI:** 10.1007/s10508-019-01620-x

**Published:** 2020-03-02

**Authors:** Mart van Dijk, Sascha B. Duken, Rosemary M. Delabre, Richard Stranz, Vincent Schlegel, Daniela Rojas Castro, Adeline Bernier, Paul Zantkuijl, Robert A. C. Ruiter, John B. F. de Wit, Kai J. Jonas

**Affiliations:** 1 Department of Work and Social Psychology, Faculty of Psychology and Neuroscience, Maastricht University, PO Box 616, 6200 MD Maastricht, The Netherlands; 2 Department of Clinical Psychology, University of Amsterdam, Amsterdam, The Netherlands; 3 Community-Based Research Laboratory, Coalition PLUS, Pantin, France; 4 AIDES, Pantin, France; 5 INSERM, IRD, SESSTIM, Sciences Economiques and Sociales de la Santé and Traitement de l’Information Médicale, Aix-Marseille Université, Marseille, France; 6 GRePS (Groupe de Recherche en Psychologie Sociale) (EA4163), Université de Lyon 2, Lyon, France; 7 Soa Aids Nederland, Amsterdam, The Netherlands; 8 Department of Social and Organizational Psychology, Utrecht University, Utrecht, The Netherlands

**Keywords:** HIV prevention, PrEP, Men who have sex with men (MSM), Sexual orientation

## Abstract

Despite increased availability of pre-exposure prophylaxis (PrEP), PrEP uptake has remained low. To promote uptake, factors related to PrEP interest among relevant target populations warrant investigation. The aim of this study was to provide an analysis of PrEP interest among men who have sex with men (MSM) in the Netherlands, while taking study recruitment strategies into account. We recruited 154 MSM from an LGBT research panel (AmsterdamPinkPanel) and 272 MSM from convenience sampling. Both samples were part of the Flash! PrEP in Europe Survey and were compared on their PrEP interest, usage intentions, and sexual behavior. We conducted logistic regression analyses to discover variables associated with PrEP interest and intentions. Participants from the AmsterdamPinkPanel were less likely to use PrEP, had less knowledge of PrEP, and were less interested in PrEP than participants from convenience sampling. Significant covariates of PrEP interest were being single, more prior PrEP knowledge, sexual risk behaviors, such as not having used a condom during last sex and having ever used drugs in a sexual context, and not participating in the AmsterdamPinkPanel. Adding the recruitment strategy to the regression increased explained variance on top of predictors already described in the literature. Increased sexual risk behavior is related to increased PrEP interest and it helps to identify PrEP target groups. Recruitment strategies have a substantial impact on findings regarding PrEP interest and usage intentions. This study emphasizes the importance of using multiple strategies for recruiting participants to obtain a more comprehensive view of MSM’s attitudes toward PrEP.

**Electronic supplementary material:**

The online version of this article (10.1007/s10508-019-01620-x) contains supplementary material, which is available to authorized users.

## Introduction

Pre-exposure prophylaxis (PrEP) is an effective biomedical intervention to prevent HIV infection among HIV-negative individuals (Grant et al., 2010, 2014; Liu et al., 2013; McCormack et al., 2016; Molina et al., 2015). In the past years, the availability and accessibility of PrEP have improved. Four years after the approval of the Food and Drug Administration (FDA) in the U.S. in 2012 (Roehr, 2012), PrEP became formally available in Europe based on the approval of the European Medicines Agency (EMA) in 2016 (European Medicines Agency, 2016). Moreover, in many countries, PrEP has become more affordable through the inclusion of PrEP coverage in health insurance, such as in Belgium and Portugal (NAM Aidsmap, 2017), and the introduction of cheaper, generic versions of PrEP in some European countries (PrEPnu, 2017).

Despite the increased accessibility of PrEP, PrEP uptake has remained low, even among men who have sex with men (MSM) who are primary candidates for PrEP (Parsons et al., 2017). MSM may underestimate their risk of getting HIV, and may therefore see themselves as not requiring PrEP (Blumenthal et al., 2019; Parsons et al., 2017). Other barriers for PrEP uptake may include medical mistrust and anticipated stigma from sex partners (Biello et al., 2017; Cahill et al., 2017; Golub, 2018). To promote PrEP uptake, it is essential to further examine factors related to interest in PrEP in populations for whom PrEP implementation should be fostered.

Previous studies have shown substantial variation in PrEP interest. MSM at higher risk of getting HIV, because of a history of sexually transmitted infections (STIs) or multiple sex partners, were found to be more interested in taking PrEP (Bil et al., 2015; Golub, Kowalczyk, Weinberger, & Parsons, 2010; Yang et al., 2013). Further, some studies reported that younger MSM were more interested in taking PrEP (Aghaizu et al., 2013; Barash & Golden, 2010), while other studies found that older MSM were more interested in taking PrEP (Yang et al., 2013); some studies did not find a relation between age and PrEP interest (Bil et al., 2015; Holt et al., 2012).

Variation in PrEP interest between studies can at least in part be explained by variation in how the question was formulated. Some studies assessed willingness to use PrEP (Barash & Golden, 2010; Golub, Gamarel, Rendina, Surace, & Lelutiu-Weinberger, 2013; Grov, Rendina, Whitfield, Ventuneac, & Parsons, 2016), while other studies combined measures of willingness to use PrEP and intention to use PrEP into one variable (Bil et al., 2015; Holt et al., 2012). Importantly, health risk research found that willingness and intention were related, but independent constructs. According to the prototype/willingness model, intentions are more reflective thoughts in order to achieve a particular goal state, while willingness does not involve goal states, plans, or instrumental actions (Gibbons, Gerrard, Ouellette, & Burzette, 1998). Consequently, research has also found a clear distinction between willingness to use PrEP and intention to use PrEP (Rendina, Whitfield, Grov, Starks, & Parsons, 2017); MSM with a high intention to use PrEP had a lower education level, a lower income, a younger age, higher beliefs in the effectiveness of PrEP, saw themselves more often as appropriate candidates for PrEP, and felt greater partner pressure for condom nonuse, compared to MSM with a high willingness to use PrEP.

In addition, different recruitment strategies might also explain part of the variation in PrEP interest within MSM samples. For instance, in a cohort study in The Netherlands in 2012, 13.5% of MSM had a high intention to use PrEP and 60% had a medium intention to use PrEP (Bil et al., 2015), while an online study in the same period found a much lower intention to use PrEP (47.8%; Frankis, Young, Flowers, & McDaid, 2016). Differences in PrEP use and PrEP interest were also found in a study that directly compared different recruitment strategies: PrEP use and PrEP interest were higher among clients of an HIV/STI testing location compared to an online MTurk sample (Beymer, Holloway, & Grov, 2018).

The aim of the current study was to provide an in-depth analysis of PrEP interest among MSM in the Netherlands, while taking recruitment strategies into account. Our first research question was what the PrEP knowledge, PrEP use, PrEP interest, and PrEP usage intentions were among MSM in the Netherlands. Next, we investigated covariates of interest in PrEP and of intention to use PrEP. Our second research question was whether participants that were solicited via different recruitment strategies differed in PrEP interest and usage intentions. Specifically, participants in an LGBT research panel were compared to participants who were recruited online and at LGBT social venues. Finally, we compared characteristics of participants recruited through the LGBT research panel with LGBT research panel members not participating in this study to shed further light on underlying differences.

## Method

### Participants

Dutch participants for the Flash PrEP in Europe (FPIE) survey were recruited via two recruitment strategies: (1) via an LGBT research panel (AmsterdamPinkPanel), or (2) via convenience sampling, through gay dating apps and websites (Hornet and PlanetRomeo), LGBT or MSM-related websites (for example, gay.nl), and 5000 flyers distributed in LGBT-themed bars, cafés, saunas, and STI clinics in June and July 2016. This provided the opportunity to compare sampling strategies, as FPIE participants stemming from the AmsterdamPinkPanel could be identified.

The AmsterdamPinkPanel is a partnership between COC Amsterdam (Dutch LGBT rights organization) and the Psychology Research Institute of the University of Amsterdam. The AmsterdamPinkPanel is a psychosocial LGBT research panel with more than 1400 members, of which 931 are MSM, with an age-range from 18 to 78 years. The members not only live in Amsterdam, but throughout the Netherlands. Sampling for the AmsterdamPinkPanel is community-driven and by self-enrollment.

Participants needed to be 18 years or older and to be HIV-negative or unaware of their HIV-status for inclusion in the current survey. Given the focus on MSM, only cisgender males were included in the analysis, defined by male gender at birth and current male gender. Participants were excluded from participation if they were HIV-positive, as PrEP is indicated only for HIV-negative individuals.

### Measures

The FPIE survey was designed by the French community-based organizations AIDES and Coalition PLUS and adjusted by input from other non-governmental organizations and academics from 12 European countries. The 82-item questionnaire was the same in all participating countries and available in the local language. Translators chose the phrasing that seemed most adequate according to the populations targeted. A back-translation in English was conducted to ensure translation accuracy. For Dutch participants, the questionnaire was offered in Dutch and English. At the start of the survey, the only information provided about PrEP was that it is an HIV-prevention tool that is available in some countries around the world. We describe the measures relevant to the current research questions below. A full description of all measures in the FPIE survey can be found in the online supplementary material. The questionnaire was created in Qualtrics and the participants could not click back to previous questions.

#### Sociodemographic Characteristics

In the FPIE survey, participants were asked to indicate their gender, age, relationship status, educational level, financial situation, country of birth, and country of residence. Gender was determined using two questions: gender at birth and current gender. Educational level was indicated by five levels, ranging from no higher education to PhD degree. Financial situation was assessed with a 6-point scale ranging from (1) “You can’t make ends meet without borrowing” to (6) “You are doing really well.”

In the annual intake survey of the AmsterdamPinkPanel, some demographic questions were asked with different response options than the questions in the FPIE survey. In the AmsterdamPinkPanel, educational level was indicated by seven levels, ranging from elementary school to university degree, and also included the options “rather not say” and “other education.” Financial situation was indicated by self-reported yearly income in one of five different categories (< €30.000, €31.000-€50.000, €51.000-€75.000, > €75.000 and “prefer not to say”).

#### Knowledge of PrEP

Participants indicated if they were aware of PrEP (yes/no). If they did, they were asked to choose the correct definition out of five options to assess their knowledge. Two of the five definitions were correct (“PrEP is a pill that greatly reduces the risk of contracting HIV. You have to take it every day.” and “PrEP is a pill that greatly reduces the risk of contracting HIV. You have to take it when you plan to have sex, before and two days after.”), and a maximum of two choices were allowed. We defined knowledge of PrEP as correct if participants chose at least one of the correct definitions and none of the incorrect definitions. After these questions, all participants were provided with information about PrEP. This stated that PrEP provides protection against HIV when the drug is sufficiently present in the blood, but that it does not provide protection against other STIs and that it should not be confused with PEP (Post-Exposure Prophylaxis).

#### PrEP Use

We asked participants if they were using PrEP. If so, they were not asked questions about interest in PrEP and intentions to use PrEP. Current PrEP users were asked how they obtained PrEP (e.g., via doctor’s prescription, online purchase, or using HIV treatments prescribed as PEP).

#### Interest in PrEP

To investigate willingness to use PrEP, we asked participants who did not use PrEP if they were interested in PrEP, using a 5-point scale, ranging from (1) definitely not interested to (5) definitely interested. Participants who indicated that they were (maybe) interested in PrEP were asked for their reasons why they were interested. They could express their agreement on the statements listed in Table 2 by using a 5-point scale, ranging from (1) strongly disagree to (5) strongly agree. Participants who indicated that they were not interested in PrEP were asked for their reasons why they were not interested. They could express their agreement on the statements listed in Table 3 by using a 5-point scale, ranging from (1) strongly disagree to (5) strongly agree. Participants could not add reasons themselves.

#### Intention to Use PrEP

Participants’ intentions to use PrEP were evaluated by asking if they intended to use PrEP when it becomes available, and before it becomes officially available within their countries’ health care system. Both questions were rated on a 5-point scale, ranging from (1) definitely having no intention to (5) definitely having the intention to use.

#### Sexual Behavior

We asked participants about the number of sex partners they had in the past 6 months and the frequency of anal sex, using a 5-point scale ranging from (1) never to (5) daily. Participants indicated if they used condoms during anal sex in the past 6 months by using a 5-point scale ranging from (1) never to (5) always.

#### Substance Use

Participants were asked if they used injectable drugs and if they used other drugs. If they responded yes to one of those questions, they were asked if they used drugs in a sexual context (yes/no).

### Data Analysis

We analyzed the data using IBM SPSS Statistics version 24. We controlled for duplicate participation by checking IP addresses and response patterns on demographics and key variables.

We used descriptive statistics to describe the sociodemographic characteristics of the sample, and undertook difference tests to compare subsamples of participants recruited through the AmsterdamPinkPanel and through convenience sampling. To investigate self-selection bias, we further assessed differences in the sociodemographic characteristics of the AmsterdamPinkPanel members who participated in FPIE with panel members who did not participate in the survey, based on intake data of the AmsterdamPinkPanel. We used descriptive statistics to describe the sample with respect to PrEP knowledge, use, interest and usage intentions, and assessed differences on these variables between AmsterdamPinkPanel participants and participants recruited through convenience sampling. For all difference tests we used analysis of variance (ANOVA) for continuous variables and chi-square tests for categorical variables. We used a multivariate analysis of variance (MANOVA) for comparing the answers on the multiple reasons for interest in PrEP, and reasons for disinterest in PrEP, between the participants recruited from the AmsterdamPinkPanel and the participants recruited through convenience sampling. Given the most common type of variables, we conducted logistic regression analyses to assess variables associated with interest in PrEP and intention to use PrEP.

## Results

### Participant Characteristics

In total, 426 participants completed the FPIE survey. The mean age of the participants was 42 years, with age ranging from 18 to 75 years (Table 1). The Netherlands was the place of birth of 363 (85.2%) participants. A bachelor degree or higher had been obtained by 290 (68.1%) participants. The average score regarding perceived financial situation was 4.29 (SD 1.24), indicating that participants perceived their financial situation between “all right” and “rather well.” The relationship status of 191 (44.8%) participants was single. We recruited 154 (36.2%) males via the AmsterdamPinkPanel and 272 (63.8%) males via convenience sampling. The AmsterdamPinkPanel participants and the participants recruited via convenience sampling differed on several sociodemographic characteristics. AmsterdamPinkPanel participants were on average older, perceived their financial situation as better, had a higher education level, and were more likely to be in a relationship (Table 1).

**Table 1:** Demographics and behaviors of MSM

	Total (*N *= 426)	Participants from AmsterdamPinkPanel (*N *= 154, 36.2%)	Participants from convenience sampling (*N *= 272, 63.8%)		*p*	*ƞ*_*p*_^2^
*M* (range/SD)				*F*(df)		
Age	42 (18–75)	51 (18–75)	36 (18–72)	133.64 (1, 424)	< .001	.24
Perceived financial situation	4.29 (1.24)	4.75 (1.09)	4.03 (1.24)	36.62 (1, 421)	< .001	.08
Number of sex partners in past 6 months	19 (36.6)	18.9 (59.9)	19.2 (23.8)	.003 (1, 268)	.95	< .01
*N* (%)				*χ*^2^(df)		
Education level						
No higher education	68 (16.2%)	17 (11.2%)	51 (19.1%)	40.81 (4)	< .001	–
Professional/vocational education	61 (14.6%)	14 (9.2%)	47 (17.6%)			
Bachelor degree	138 (32.9%)	40 (26.3%)	98 (36.7%)			
Master degree	118 (28.2%)	55 (36.2%)	63 (23.6%)			
PhD degree	34 (8.1%)	26 (17.1%)	8 (3.0%)			
Relationship status						
Single	209 (49.2%)	56 (36.4%)	153 (56.5%)	26.11 (2)	< .001	–
In a relationship	123 (28.9%)	67 (43.5%)	56 (20.7%)			
In an open relationship	93 (21.9%)	31 (20.1%)	62 (22.9%)			
Had an STI in the past 12 months	80 (20.3%)	17 (12.1%)	63 (24.8%)	9.12 (1)	.003	–
Used a condom the last time	201 (51.1%)	69 (49.3%)	132 (52.2%)	.30 (1)	.58	–
Used drugs in a sexual context	136 (33.2%)	38 (25.3%)	98 (37.7%)	6.55 (1)	.01	–

Furthermore, we could identify differences between AmsterdamPinkPanel participants and AmsterdamPinkPanel members who did not participate in the FPIE study. We invited all 931 male members of the AmsterdamPinkPanel to participate in the FPIE study, of whom 203 (21.8%) decided to participate. The survey was completed by 154 participants. To answer our question whether there was a self-selection bias in MSM who responded to a survey about PrEP among research panel members, we compared AmsterdamPinkPanel members who participated in FPIE (*n *= 203) with AmsterdamPinkPanel members who did not participate (*n *= 728) on demographic variables. We observed differences in age and income, based on intake survey data: AmsterdamPinkPanel members participating in FPIE (*M* age = 52, range 18–81) were older than AmsterdamPinkPanel members who did not participate (*M* age = 46, range 19–90; *F*[1, 926] = 29.14, *p *< .001, *ƞ*_*p*_^2^ = .031). Of those who provided information on their income (*n *= 850), AmsterdamPinkPanel members participating in FPIE had a higher income than the AmsterdamPinkPanel members who did not participate, with 19.9% of the AmsterdamPinkPanel members participating in FPIE having an income below € 31.000 compared to 33.1% of the AmsterdamPinkPanel members who did not participate (*χ*^2^[4] = 18.74, *p *< .001). AmsterdamPinkPanel members overall are well educated, with a majority (75.5%) having a Bachelor degree or higher. There were no differences in education level between the AmsterdamPinkPanel members participating in FPIE or not (*χ*^2^[6] = 5.00, *p *= .54). AmsterdamPinkPanel members participating in FPIE were less often single (36.0%) than other AmsterdamPinkPanel members (44.8%), but there was no significant difference in relationship status between the two groups (*χ*^2^[6] = 9.17, *p *= .16).

### PrEP Knowledge and PrEP Use

Out of the 426 FPIE participants, 383 (89.9%) indicated that they were aware of PrEP. Of those who were aware of PrEP, 327 participants (85.4% of those who were aware of PrEP) had correct knowledge of PrEP. Thirty-four participants (8.0%) mistook PrEP for Post-Exposure Prophylaxis (PEP). AmsterdamPinkPanel participants were less likely to have correct knowledge about PrEP than the participants recruited through convenience sampling (77.1% vs. 89.7%, *χ*^2^[1] = 10.93, *p *< .001).

Out of the 426 FPIE participants, 29 (6.6%) used PrEP. AmsterdamPinkPanel participants used PrEP less often than the participants recruited through convenience sampling (3.2% vs. 8.8%, *χ*^2^[1] = 4.82, *p *= .03). One participant received PrEP via a doctor’s prescription. The remaining 28 participants received PrEP via a research trial, such as the Dutch AMPrEP trial (Hoornenborg et al., 2019).

### Interest in PrEP

Out of 426 FPIE participants, 120 (28.2%) were definitely interested in using PrEP, and 73 (17.1%) participants were probably interested in using PrEP. There was a substantial difference in PrEP interest between participants recruited from the AmsterdamPinkPanel and the participants from convenience sampling (*χ*^2^[4] = 100.33, *p *< .001). The majority of participants recruited from the AmsterdamPinkPanel were not interested in using PrEP; 53 (36.1%) of these participants were definitely not interested and 36 (24.5%) were probably not interested. In contrast, the majority of the participants recruited through convenience sampling were interested in using PrEP; 106 (42.9%) of these participants were definitely interested and 57 (23.1%) were probably interested.

For those participants (*n *= 260) who were interested in using PrEP we assessed the reasons for their interest (Table 2). In a MANOVA, we found no significant difference in the reasons for interest in PrEP between AmsterdamPinkPanel participants and the participants recruited through convenience sampling (*F*[6, 253] = 1.76, *p *= .11, Wilk’s *Λ* = .96, *ƞ*_*p*_^2^ = .04).

**Table 2:** Mean scores on reasons for interest in PrEP for AmsterdamPinkPanel participants and the participants recruited through convenience sampling

	Participants from AmsterdamPinkPanel *M* (*SD*) *N *= 58	Participants from convenience sampling *M* (*SD*) *N* = 202	*F*(1, 258)	*p*	*ƞ*_*p*_^2^
I’d rather have condomless sex	3.76 (1.33)	3.46 (1.40)	2.16	.14	.008
I’m at risk of being infected by HIV	3.57 (1.19)	3.69 (1.10)	.58	.46	.002
I would feel safer	4.33 (1.02)	4.58 (.76)	4.23	.04	.016
I would feel less anxious	4.24 (1.10)	4.44 (.85)	2.15	.14	.008
I would feel more in control	4.17 (.96)	4.36 (.88)	1.99	.16	.008
I would have a more satisfying sex life	3.62 (1.28)	3.93 (1.22)	2.85	.09	.011

The scores are on a 5-point Likert scale, with (1) for strongly disagree and (5) for strongly agree. Please note that in a MANOVA, no significant difference was found in the reasons for interest in PrEP between AmsterdamPinkPanel participants and the participants recruited through convenience sampling (*F*[6, 253] = 1.76, *p *= .11, Wilk’s *Λ* = .96, *ƞ*_*p*_^2^ = .04)

For those participants (*n *= 119) who were not interested in using PrEP we assessed the reasons for their lack of interest (Table 3). In a MANOVA, there was no significant difference in the reasons for lack of interest in PrEP between AmsterdamPinkPanel participants and the participants from convenience sampling (*F*[10, 108] = 1.64, *p *= .10, Wilk’s *Λ* = .87, *ƞ*_*p*_^2^ = .13).

**Table 3:** Mean scores on reasons for non-interest in PrEP for AmsterdamPinkPanel participants and the participants recruited through convenience sampling

	Participants from AmsterdamPinkPanel *M* (*SD*) *N *= 82	Participants from convenience sampling *M* (*SD*) *N *= 37	*F*(1, 117)	*p*	*ƞ*_*p*_^2^
I don’t want to take medication every day	4.38 (1.22)	3.84 (1.46)	4.39	.04	.036
I don’t want to pay for PrEP	2.67 (1.31)	2.65 (1.27)	.01	.93	< .001
I’m worried about the side-effects	3.93 (1.15)	3.70 (1.27)	.91	.34	.008
I’m afraid of being seen in a negative light if I take PrEP	2.35 (1.26)	2.11 (1.13)	1.03	.31	.009
I don’t believe it works	2.09 (1.19)	2.57 (1.30)	3.95	.05	.033
I’m worried of getting other STIs	3.26 (1.52)	3.51 (1.54)	.72	.40	.006
I don’t need to change how I protect myself	4.39 (.94)	4.27 (1.10)	.37	.54	.003
I don’t think I’m at risk of being infected by HIV	3.77 (1.18)	3.19 (1.41)	5.43	.02	.044
I don’t want to undergo regular medical check-ups	2.91 (1.44)	2.81 (1.39)	.14	.71	.001
I’m worried that I might use condoms less often	2.56 (1.43)	2.65 (1.47)	.09	.76	.001

The scores are on a 5-point Likert scale, with (1) for strongly disagree and (5) for strongly agree. Please note that in a MANOVA, no significant difference was found in the reasons for lack of interest in PrEP between AmsterdamPinkPanel participants and the participants from convenience sampling (*F*[10, 108] = 1.64, *p *= .10, Wilk’s *Λ* = .87, *ƞ*_*p*_^2^ = .13)

### Intentions to Use PrEP

Regarding intention to use PrEP when it becomes officially available within the countries’ health care system, we saw the same distribution of scores as for PrEP interest. Out of 426 FPIE participants, 119 (27.9%) participants definitely had the intention to use PrEP when it becomes available and 81 (19.0%) participants probably had the intention to use PrEP when it becomes available. The participants recruited through convenience sampling had a higher intention than AmsterdamPinkPanel participants (*χ*^2^[5] = 100.74, *p *< .001). Of the participants recruited through convenience sampling, 39.9% definitely and 28.6% probably had the intention to use PrEP when it becomes available, compared to 13.4% and 6.7% of AmsterdamPinkPanel participants.

The intention to use PrEP before it becomes officially available was substantially lower than the intention to use PrEP when it becomes available. Only 26 (6.1%) participants definitely and 37 (8.7%) participants probably had the intention to use PrEP before it becomes available. Again, participants recruited through convenience sampling had a higher intention than AmsterdamPinkPanel participants (*χ*^2^[5] = 55.69, *p *< .001). Of participants recruited through convenience sampling, 9.3% definitely, and 12.1% probably had the intention to use PrEP before it becomes available, compared to 2.0% and 4.7% of AmsterdamPinkPanel participants.

### Factors Associated with PrEP Interest and Intention to Use PrEP

Table 4 shows the outcomes of the logistic regression of correlates of interest in PrEP. Significant multivariate correlates of PrEP interest were being single (aOR = .31, 95% CI .17–59, *p *< .001), having correct prior PrEP knowledge (aOR = 1.96, 95% CI 1.05–3.69, *p *= .04), not having used a condom for last sex (aOR = 2.00, 95% CI 1.17–3.42, *p *= .01), having ever used drugs in a sexual context (aOR = 2.68, 95% CI 1.52–4.73, *p *= .001), and not being a member of the AmsterdamPinkPanel (aOR = .18, 95% CI .10–.34, *p *< .0001). Adding the variable “being a member of the AmsterdamPinkPanel” to the regression model increased the proportion of explained variance by eight percent from *R*^2^ = .29 (*χ*^2^[12, *N* = 358] = 87.74, *p *< .001) to *R*^2^ = .37 (*χ*^2^[13, *N* = 358] = 117.38, *p *< .001).

**Table 4:** Multivariable logistic regression examining correlates of interest in PrEP

	B	S.E.	*p*	aOR	95% CI
Age	.01	.01	.55	1.00	.99–1.03
Education level					
No higher education	Ref				
Professional qualification	.59	.45	.19	1.81	.65–4.38
Bachelor	.08	.39	.83	1.08	.51–2.31
Master	.47	.41	.26	1.60	.71–3.59
PhD	−.17	.64	.79	.84	.24–2.94
Financial situation	−.15	.11	.18	.86	.69–1.07
Relationship status					
Single	Ref				
In a relationship	−1.16	.32	< .001	.31	.17–.59
In an open relationship	.07	.34	.83	1.08	.55–2.10
STI in past 12 months*	.42	.36	.24	1.53	.76–3.08
PrEP knowledge*	.68	.32	.04	1.96	1.05–3.69
Not used a condom the last time*	.69	.27	.01	2.00	1.17–3.42
Used drugs in a sexual context*	.99	.29	.001	2.68	1.52–4.73
Participant of AmsterdamPinkPanel*	−1.71	.33	< .001	.18	.10–.34

*The reference category for these variables is “no”

*χ*^2^[13, *N* = 358] = 117.38, *p *< .001, Nagelkerke *R*^2^ = .37

Similarly, we conducted a logistic regression analysis regarding correlates of intention to use PrEP. The same multivariate correlates as for PrEP interest were found for intention to use PrEP, except for having correct prior PrEP knowledge (aOR = 1.40, 95% CI .75–2.62, *p *= .29). Significant multivariate correlates of intention to use PrEP were being single (aOR = .42, 95% CI .23–.79, *p *= .007), not having used a condom for last sex (aOR = 1.88, 95% CI 1.09–3.23, *p *= .02), having ever used drugs in a sexual context (aOR = 2.90, 95% CI 1.62–5.17, *p* < .001), and not being a member of the AmsterdamPinkPanel (aOR = .13, 95% CI .07–.25, *p *< .001). Adding the variable “being a member of the AmsterdamPinkPanel” to the regression model increased the proportion of explained variance by 12 percent from *R*^2^ = .27 (*χ*^2^[12, *N *= 356] = 79.65, *p *< .001) to *R*^2^ = .39 (*χ*^2^[13, *N* = 356] = 121.91, *p *< .001).

## Discussion

The aim of this study was to provide an in-depth analysis of PrEP interest of MSM while taking recruitment strategies into account. It is important to examine factors related to interest in PrEP to be able to identify who can be targeted to increase PrEP uptake, because PrEP uptake has generally remained low, even among individuals with an elevated risk of an HIV infection (Parsons et al., 2017). Moreover, taking recruitment strategies into account is important since previous studies making use of different recruitment strategies have shown substantial variation in PrEP interest (Beymer et al., 2018; Bil et al., 2015; Frankis et al., 2016; Holt et al., 2012; Yang et al., 2013). For the current study, we recruited participants in two ways: via an LGBT research panel (AmsterdamPinkPanel) and via convenience sampling.

We found that recruitment strategies had a substantial impact on findings regarding interest in PrEP and intention to use PrEP. In particular, in the regression analysis, we found that adding recruitment strategy as a covariate substantially increased the proportion of explained variance of interest in PrEP and intention to use PrEP. We found that MSM participants recruited through the AmsterdamPinkPanel were less interested in PrEP and had a lower intention to use PrEP than MSM participants who were recruited via convenience sampling. Also, AmsterdamPinkPanel participants were older, more educated, wealthier, and more often in a relationship compared to the participants from convenience sampling. Previous studies showed mixed results for the influence of sampling strategies on findings regarding interest in PrEP. Beymer et al. (2018), for example, reported higher interest in PrEP among visitors to an STI clinic compared to online participants, while Ferrer et al. (2016) reported lower interest among visitors to an STI clinic compared to online participants. Overall, the pattern of results across studies is diverse, confirming our notion that recruitment strategies and sample characteristics can play a major role in explaining different findings.

Overall, we found that 89.9% of the participants already knew what PrEP is. This is a large proportion compared to earlier studies (Bil et al., 2015; Frankis et al., 2016; Grov et al., 2016), suggesting that knowledge of PrEP is increasing over the years. Despite the high level of PrEP knowledge, we found low actual use of PrEP (6.6%). This is comparable to findings of other studies in Europe (Bourne et al., 2019), and globally (Kamitani et al., 2018), and likely reflects the early stages of PrEP implementation. Nevertheless, about half of the participants (45.3%) were interested in taking PrEP, and a similar number of participants (46.9%) had the intention to use PrEP when available. This is in line with the majority of earlier studies, reporting a willingness to use PrEP among about 50% of participants, as described in a review by Young and McDaid (2014). Covariates of interest in PrEP were being single, having correct prior PrEP knowledge, not having used a condom for last sex, having ever used drugs in a sexual context, and not being participant of the AmsterdamPinkPanel. The same covariates were found for intention to use PrEP, except for the covariate having correct prior PrEP knowledge. In contrast with the notion that there is a clear distinction between willingness to take PrEP and intention to take PrEP (Rendina et al., 2017), we did not encounter this distinction in our results. Because PrEP had only limited availability in the Netherlands at the time of our study, PrEP use was fairly distant for most MSM and, therefore, it may not have been possible to find this fine distinction between interest in PrEP and intention to use PrEP, as was found in the U.S.-based study of Rendina et al. (2017).

To investigate the possibility of a self-selection bias in MSM who respond to a survey about PrEP, we compared AmsterdamPinkPanel members who participated in FPIE with AmsterdamPinkPanel members who did not participate. We found that MSM from the AmsterdamPinkPanel who participated in the FPIE survey were older and more affluent than MSM from the AmsterdamPinkPanel who did not participate. This finding is surprising since we expected that younger MSM would be more likely to respond to the questionnaire, because they are more interested in taking PrEP according to previous studies (Holt et al., 2012). However, the majority of the AmsterdamPinkPanel participants (60.6%) were not interested in using PrEP, which may have influenced their motivation to take part in the survey and to voice their views.

A limitation of our study is that, while we recruited participants via different strategies, inclusion was based on convenience sampling. Random population sampling strategies, in which each individual in the population has the same probability of being included, seem to be less affected by self-selection bias than nonprobability samples (Meyer & Wilson, 2009), and LGBT probability samples are found to differ from LGBT participants in nonprobability community samples. LGBT nonprobability community participants were younger, had more often an exclusive same-sex orientation, and were more open about their sexual orientation (Kuyper, Fernee, & Keuzenkamp, 2016). They also reported more high-risk sexual behavior (Dodds, Mercer, Mercey, Copas, & Johnson, 2006; Evans, Wiggins, Mercer, Bolding, & Elford, 2007). However, probability samples for LGBT participants are expensive to establish, and most studies on PrEP use and PrEP interest are based on nonprobability samples. The LGBT population makes up a small fraction of the general population, requiring the recruitment or screening for inclusion of many people ineligible for participation to be able to include a sufficient amount of LGBT participants (Meyer & Wilson, 2009). However, a recent study, conducted in the U.S. in 2016, made use of data from a probability sample to examine PrEP use and familiarity with PrEP among MSM (Hammack, Meyer, Krueger, Lightfoot, & Frost, 2018). They found that 4.1% of the respondents had used PrEP, and that 59.8% was familiar with PrEP. This level of PrEP use is comparable to the online nonprobability samples in the study of Beymer et al. (2018), who conducted the study in roughly the same period (2015–2016).

A further limitation is that we could only compare AmsterdamPinkPanel participants with the participants from convenience sampling, but could not make further distinctions within the latter group. We could not track the recruitment source of the convenience sampling participants, because this was not a primary goal of the FPIE survey. For example, we could not compare participants recruited through gay dating apps with participants recruited through gay social venues. This is a drawback as it is expected that MSM who use gay dating apps have different sexual behaviors than MSM who are not using such apps (Lewnard & Berrang-Ford, 2014). Another limitation is that the results were based on participants’ self-reports, which may be affected by reporting bias, as opposed to clinical data such as measuring intracellular PrEP drug levels. However, as participants filled out the questionnaire anonymously, social desirability bias is expected to be limited.

For future research, we recommend that researchers not only focus on obtaining an MSM sample per se, but to carefully consider the characteristics of the sample they recruit, and how this can influence their findings. Having clear inclusion and exclusion criteria, and drawing on different sample sources and recruitment strategies, will help to identify and address potential sampling biases. A strength of our study is that we could compare two samples that were recruited at the same time, eliminating the influence of timing. This is important because PrEP is relatively new and community awareness and accessibility are increasing.

In conclusion, while findings show differences between samples according to recruitment strategies, overall findings suggest that PrEP knowledge is high among participating MSM, but PrEP use is low. About half of the participants were interested in using PrEP, and findings regarding covariates of interest in PrEP and intention to use PrEP provide important directions for the promotion of PrEP. Promotional activities may in particular target MSM who are single, do not always use condoms, and use drugs in a sexual context. Promoting PrEP among these MSM may be especially pertinent to increase PrEP uptake.

## Electronic supplementary material

Below is the link to the electronic supplementary material.

Supplementary material 1 (DOCX 36 kb)
