# Supplementary material for: PrEP Interest Among Men Who Have Sex with Men in the Netherlands: Covariates and Differences Across Samples
Source: Arch Sex Behav. 2020 Mar 2;49(6):2155–64. doi: 10.1007/s10508-019-01620-x (PMC7316842; doi:10.1007/s10508-019-01620-x)
Supplement: Supplementary file 1 — Supplementary material 1 (DOCX 36 kb) [file 10508_2019_1620_MOESM1_ESM.docx]

**Flash! PrEP in Europe**

Hello,

 A new prevention tool for HIV exists and your input will help better understand its usefulness across Europe.

 The tool is **PrEP** and it is already available in several countries worldwide (France, United-States, Canada, Kenya, Thaïland and South Africa amongst others). The survey is being conducted simultaneously in eleven European countries. We need your answers to be able to efficiently represent and defend your needs concerning PrEP.

 It will take you between 10 and 20 minutes to fill out the questionnaire. Your participation is voluntary and anonymous. It is very important for us that you answer the whole questionnaire, if possible.

 The results of the survey will be available (list of websites below) in the second half of 2016. It is also possible for you to leave us your e-mail address at the end of the survey so that we can send you the findings.
  
 We hope you take part in the survey and please feel free to suggest it to people you know.


**The Flash PrEP Europe Study Group.**

**IMPORTANT** **INFORMATION**

 **Informed consent**
 You are about to take part in a scientific study that will allow researchers to better understand current sexual health issues around HIV prevention and how to move to address them in the key populations. The ultimate goal is to advocate to improve sexual health of those most exposed to being infected with HIV.
 Before you begin, it is important that you know about the conditions and procedures of this study. Please read carefully the information below.

 **Participant criteria**
 Participants need to be 18 years or older and be HIV negative.

 **Goal of this research**
 The goal of this research is to better understand knowledge, attitudes towards, willingness to take and actual (informal and less official) use of PrEP to inform sexual health policy making across Europe.

 **Instructions and procedure**
 You will be asked to tell us about your personal experiences regarding your sexual and drug taking behaviours and to answer some general questions about yourself.

 **Participation is voluntary**
 Your participation in this study is completely voluntary. Without giving a reason, you can quit at any moment. Alternatively, if you wish to withdraw your agreement and the information you've contributed, you can do so for up to 24 hours and your data will be removed from our system.

 **Risks**
 There are neither health nor security risks related to this study. No extra liability insurance has been put in place by UvA. 

 **Privacy**
 Your data will be used solely for the purpose of research, analysis, and presentation. This means that your data and personal information will be treated completely anonymously, and all IP addresses will be removed prior to analysis. Data may be shared with other researchers, but only in a fully anonymous format.

 **Further information**
 If you would like to know more about this study, please contact the principal investigators at prepineurope@gmail.com

 **Coordination committee members**: Daniela Rojas Castro, Adeline Bernier, Vincent Schlegel, Kai Jonas & Richard Stranz.

 **Ethical approval**
 This survey has obtained the prior approval of the Ethics Committee of the University of Amsterdam (2016-SP-7030).
 Should you have any complaints about this study, please contact the social psychology representative of the Ethics Committee of the University of Amsterdam.

 **Informed consent**
 By continuing, you agree that the terms and conditions of this research are clear to you and that you voluntarily decide to take part in this study.

I agree to the above conditions and confirm that I am 18 years or older

- I am 18 years or older and I want to participate in the survey
- I am younger than 18, or I do not want to participate in the survey

|  |
| --- |

Before we start with the survey, we would like to know your HIV status.

- I'm HIV-positive
- I'm HIV-negative
- I don't know my HIV status
- I don't want to provide this information

Do you already know what PrEP is?

- Yes
- No

What, from your point of view, is the best description of PrEP? (*maximum 2 choices*)

- PrEP is a pill that you can use after you think you have been at risk of being infected by HIV.
- PrEP is a pill that greatly reduces the risk of contracting HIV. You have to take it every day.
- PrEP is a pill that greatly reduces the risk of contracting HIV and Sexually Transmitted Infections (STIs).
- PrEP is a pill that greatly reduces the risk of contracting HIV. You have to take it when you plan to have sex, before and two days after.
- PrEP is a pill that greatly reduces the risk of contracting HIV. You have to take it once or twice a week.

How did you learn this information? *(more than one answer possible)*

- From a doctor/medical personnel
- From a non-governmental organisation (NGO/charity)
- From one or more friends
- From one or more HIV-positive persons
- From the mainstream media (newspapers, Internet, etc.)
- From the community media (specialised websites, blogs, etc.)
- From social media (Facebook, Twitter, etc.)
- From scientific papers/websites
- Other (please specify) ________________________________________________

***PrEP*** *(pre-exposure prophylaxis) is the use of an HIV/antiretroviral drug by an HIV-negative individual for preventive purposes, that is, to prevent HIV infection. Truvada® is the main drug used as PrEP.*

 Studies have shown that it can be taken on a daily basis or continuous regimen (as in PROUD or iPrex trials). Other studies have shown that it can be taken intermittently, *before and after sex, on an on-demand or event-driven basis (IPERGAY study).*
  
*Currently available data show that PrEP users in both situations above are sufficiently protected against HIV if the drug is present in their blood. PrEP does not provide protection against other sexually transmitted infections (STIs).

 PrEP is not to be confused with* ***PEP*** *(post-exposure prophylaxis) which is a combination of HIV/antiretroviral drugs to prevent HIV* ***after*** *exposure to the virus.

 Requests have been made to European health authorities to permit the prescribing of Truvada® in the best possible conditions (supervision, safety, coverage and evaluation).*

|  |
| --- |

Are you already using PrEP?

- Yes
- No

Please answer the following question:

|  | No, definitely not | No, probably not | Maybe | Yes, probably | Yes, definitely |
| --- | --- | --- | --- | --- | --- |
| Based on your life and what you know, would PrEP meet your HIV prevention needs? |  |  |  |  |  |

|  |
| --- |

About your interest in using PrEP:

|  | No, definitely not | No, probably not | Maybe | Yes, probably | Yes, definitely |
| --- | --- | --- | --- | --- | --- |
| Are you interested in using PrEP? |  |  |  |  |  |

|  |
| --- |

About your intention to use PrEP:

|  | No, definitely not | No, probably not | Maybe | Yes, probably | Yes, definitely | Not applicable |
| --- | --- | --- | --- | --- | --- | --- |
| Is it your intention to use it **if and when** it becomes officially available in your country? |  |  |  |  |  |  |
| Is it your intention to use it **before** it becomes officially available in your country? |  |  |  |  |  |  |

|  |  |
| --- | --- |

In your opinion, where would be the best places to prescribe PrEP? *(maximum 2 choices)*

- At a hospital
- At a community health center, a checkpoint or LGBT/HIV clinic
- At an STI clinic
- At a General Practitioner's
- At a NGO/charity
- I don't have any preference

|  |
| --- |

What is the maximum amount, if anything, that you would be willing to pay for a batch of 30 pills? (please enter only digits)

________________________________________________________________

In which currency did you enter this amount?

- Euro
- CHF
- Danish Crone
- Romanian Leu
- GBP
- Other ________________________________________________

|  |
| --- |

Do you think it should be free of charge/covered by a health insurance for persons who use/need it?

- Yes
- No
- In part, people who use it should pay some of the cost
- I don't know

Please answer the following two questions:

|  | No, definitely not | No, probably not | Maybe | Yes, probably | Yes, definitely |
| --- | --- | --- | --- | --- | --- |
| Do you think PrEP should be made officially available backed up by a comprehensive prevention package (regular HIV testing, STI testing and treatment, peer support, etc.)? |  |  |  |  |  |
| Would you go and get PrEP **if and when** such a prevention package is officially available in your country? |  |  |  |  |  |

|  |
| --- |

How would you react if a sexual partner (principal or casual) said they were on PrEP?

|  | Extremely unlikely | Somewhat unlikely | Neither likely nor unlikely | Somewhat likely | Extremely likely |
| --- | --- | --- | --- | --- | --- |
| I would refuse to have sex with them |  |  |  |  |  |
| I would ask them to use a condom |  |  |  |  |  |
| I would have condomless sex with them |  |  |  |  |  |

Why are you interested in taking PrEP?

|  | Strongly disagree | Somewhat disagree | Neither agree or disagree | Somewhat agree | Strongly agree |
| --- | --- | --- | --- | --- | --- |
| I'd rather have condomless sex) |  |  |  |  |  |
| I'm at risk of being infected by HIV |  |  |  |  |  |
| I would feel safer |  |  |  |  |  |
| I would feel less anxiou |  |  |  |  |  |
| I would feel more in control |  |  |  |  |  |
| I would have a more satisfying sex life |  |  |  |  |  |

Would you still be interested in taking PrEP...

|  | Extremely unlikely | Somewhat unlikely | Neither likely nor unlikely | Somewhat likely | Extremely likely |
| --- | --- | --- | --- | --- | --- |
| Even if you had to pay for it? |  |  |  |  |  |
| Even if it meant taking medication everyday? |  |  |  |  |  |
| Even though there are side-effects? |  |  |  |  |  |
| Even if you have to undergo regular medical check-ups? |  |  |  |  |  |
| Even if you had to go to the hospital to get it? |  |  |  |  |  |

Why are you not interested in taking PrEP?

|  | Strongly disagree | Somewhat disagree | Neither agree nor disagree | Somewhat agree | Strongly agree |
| --- | --- | --- | --- | --- | --- |
| I don't want to take medication every day |  |  |  |  |  |
| I don't want to pay for PrEP |  |  |  |  |  |
| I'm worried about the side-effects |  |  |  |  |  |
| I'm afraid of being seen in a negative light if I take PrEP |  |  |  |  |  |
| I don't believe it works |  |  |  |  |  |
| I'm worried of getting other STIs |  |  |  |  |  |
| I don't need to change how I protect myself |  |  |  |  |  |
| I don't think I'm at risk of being infected by HIV |  |  |  |  |  |
| I don't want to undergo regular medical check-ups |  |  |  |  |  |
| I'm worried I might use condoms less often |  |  |  |  |  |

Your gender at birth was:

- Male
- Female
- Other
- I prefer not to say

Your gender now is:

- Male
- Female
- Non-binary/other
- I prefer not to say

|  |
| --- |

How old are you?

________________________________________________________________

What country were you born in?

________________________________________________________________

|  |
| --- |

In which country do you live at the moment?

________________________________________________________________

At the moment, you live in:

- A very large city (population of 1 million or more)
- A large city (population of 500,000 to 1 million)
- A medium-sized city (population of 100,000 to 500,000)
- A small city (population of 10,000 to 100,000)
- A town (population under 10,000)

|  |
| --- |

What is your current relationship status?

- Single
- Having dates
- In a relationship
- In an open relationship

Do you have any children?

- Yes
- No

How many years did you go to school?

- 0-9 years
- 10 years or more

Are you attending or have you finished higher education?

- Yes
- No

What studies are you currently enrolled in or what is the highest qualification you obtained?

- Professional/Vocational qualification
- Bachelor's degree or equivalent
- Master's degree or equivalent
- PhD/Doctorate or equivalent

Currently, how would you say you are doing financially?

- You can't make ends meet without borrowing
- You are having problems making ends meet
- You are getting by but have to be careful
- Things are all right
- You are doing rather well
- You are doing really well

How satisfied are you?

|  | Extremely dissatisfied | Somewhat dissatisfied | Neither satisfied nor dissatisfied | Somewhat satisfied | Extremely satisfied |
| --- | --- | --- | --- | --- | --- |
| Considering your life in general, would you say that you are: |  |  |  |  |  |
| Concerning your sex life, would you say that you are: |  |  |  |  |  |

We would like to know what you think about your own life, and how you look back at your own past. You will see a number of statements below. We would like know if you agree with them or not.

|  | Strongly disagree | Somewhat disagree | Neither agree nor disagree | Somewhat agree | Strongly agree |
| --- | --- | --- | --- | --- | --- |
| 1) What happened in the past does not matter much to me |  |  |  |  |  |
| 2) I mostly focus on the things that happen now |  |  |  |  |  |
| 3) I mostly focus on the future |  |  |  |  |  |
| 4) I often think about what could have been done differently in the past |  |  |  |  |  |
| 5) There are many events in the past where wished they had taken a different turn or produced a different outcome |  |  |  |  |  |
| 6) If I want to achieve a goal, I reflect what I could have done differently in the past |  |  |  |  |  |
| 7) I believe we constantly have to adjust our behavior to reach our goals |  |  |  |  |  |
| 8) I believe that we are mostly driven by fate |  |  |  |  |  |
| 9) There are other forces than myself that determine what happens to me |  |  |  |  |  |
| 10) I often think that things in the past could have been turned out better |  |  |  |  |  |
| 11) I often think about what I should have done in the past |  |  |  |  |  |
| 12) I often think about what I should not have done in the past |  |  |  |  |  |

Are you taking products to increase your muscle mass and performance?

- Yes, creatine products
- Yes, protein (whey)
- Yes, both creatine and protein (whey)
- No

Have you had vaginal and/or anal sex?

- Yes, in the past 6 months
- Yes, more than 6 months ago
- No, never

How old were you when you first had sex? *Please enter a number only, for example 15*.

________________________________________________________________

Are you sometimes attending one of the following events?

More than one answer is possible.

- Circuit parties (e.g. Rapido, La Demence, Circuit Barcelona, gCircuit)
- Gay cruiseship trips
- Sex parties/saunas
- Private sex parties

**In the past 6 months**, you have had sex with: *(more than one answer possible)*

- Men
- Women
- Transgenders/Transsexuals

Currently, how do you rate your risk of becoming infected with....?

|  | Low | Rather low | Average | Rather high | High |
| --- | --- | --- | --- | --- | --- |
| HIV |  |  |  |  |  |
| An STI (gonorrhea, chlamydia, syphilis, etc.) |  |  |  |  |  |

Do you have a main sex partner (e.g. boyfriend/girlfriend or husband/wife) at the moment?

- Yes
- No

Your main sex partner is:

- HIV-negative
- HIV-positive
- I don't know their HIV status
- I don't want to say it

Do you know what the viral load of your main sex partner is?

- I don't know what a viral load is
- Yes, their viral load is detectable
- Yes, their viral load is undetectable
- No, I don't know what their viral load is

**In the past 6 months**, have you had sex with partners other than your main sex partner?

- Yes
- No

Were they:

- One-off, casual encounters
- Regular sex partners (e.g. fuck buddies or sex friends)

How many different sex partners have you had **in the past 6 months,** excluding your main partner, if you have one?
 *[Give an approximate figure if you don't know the exact number]*

________________________________________________________________

**In the past 6 months**, how often did you have:

|  | Never | Less than once a month | Once a month or more | Once a week or more | Daily |
| --- | --- | --- | --- | --- | --- |
| Vaginal sex |  |  |  |  |  |
| Anal sex |  |  |  |  |  |

**In the past 6 months**, excluding your main partner (if you have one), how often have you used the following methods with these partners to reduce the risk of HIV infection?

|  | Never | Rarely | From time to time | Nearly always | Always | Not applicable |
| --- | --- | --- | --- | --- | --- | --- |
| I use a condom for vaginal sex |  |  |  |  |  |  |
| I use a condom for anal sex |  |  |  |  |  |  |
| I choose partners who say they are HIV-negative |  |  |  |  |  |  |
| I adapt the sex I have depending on my partner's HIV-statu |  |  |  |  |  |  |
| I have sex with HIV-positive partners who say their viral load is undetectable |  |  |  |  |  |  |

Did you use a condom when you last had vaginal and/or anal sex?

- Yes
- No

Have you ever received money, goods or drugs in exchange for sex?

- Yes, in the past 12 months
- Yes, more than a year ago
- No, never

Have you ever injected yourself or been injected with drugs?

- Yes, in the past 12 months
- Yes, more than a year ago
- No, never

Did you inject or were you injected with drugs in a sexual context? (e.g. slam)

- Yes
- No

Have you ever taken drugs (other than by injection)?

- Yes, in the past 12 months
- Yes, more than a year ago
- No, never

Did you take drugs in a sexual context? (e.g. chemsex parties, smoking crystal meth etc.)

- Yes
- No

Have you ever had sex against your will because of verbal, physical or any other form of pressure?

- Yes
- No

Have you ever been tested for HIV?

- Yes, in the past 12 months
- Yes, more than a year ago
- No, never

How many HIV tests have you had **in the past 12 months?**

- 1
- 2
- 3
- 4
- 5 or more

Have you ever been diagnosed with a sexually transmitted infection (other than HIV)?

- Yes, in the past 12 months
- Yes, more than a year ago
- No, never
- I don't know

How many times have you been diagnosed with an STI **in the past 12 months**?

- 1
- 2
- 3
- 4
- 5 or more

Have you ever used post-exposure prophylaxis for HIV (PEP or "emergency treatment") directly after being exposed to HIV? (*and you didn't use it as PrEP*)

- Yes, more than once during the past 12 months
- Yes, once during the past 12 months
- Yes, more than a year ago
- No, never

At the moment, are you participating in a study in which PrEP is provided?

- Yes
- No

You said that you are participating in a study in which PrEP is provided. Is this the AmPrEP study of the GGD Amsterdam?

- Yes
- No

In which type of study are you?

- I am supposed to take PrEP every day (daily use)
- I should take PrEP when I plan to have sex (intermittent use)

You said that you are supposed to take PrEP daily. Do you really do that?

- Yes, I take a pill every day
- No, I skip some days

What are you doing with the PrEP pills that are left over?
More answers are possible!

- I give them back to the GGD
- I am not getting new ones until I really need some
- I give them to my sex partners (free)
- I give them to my friends (free)
- I give them to strangers (free)
- I sell them to my sex partners (please enter below how much you ask per pill) ________________________________________________
- I sell them to my friends (please enter below how much you ask per pill) ________________________________________________
- I sell them to strangers (please enter below how much you ask per pill) ________________________________________________

Have you ever taken medication (**PrEP**) to prevent an HIV infection?

- Yes
- No

| Page Break |  |
| --- | --- |

For how many months have you been using PrEP?

- I'm no longer using it at the moment
- Less than 3 months
- 3-6 months
- 7-12 months
- For more than a year (please indicate length of time): ________________________________________________

What tablet dosing regimen do you (or did you) usually use ?

- Every day (continuous regimen)
- Before and after sex or on-demand (intermittent regimen)
- Other dosing regimen (please specify): ________________________________________________

Which drug are you using or did you use for PrEP ? *(more than one answer possible)*

- Truvada®
- Generic Truvada®
- Other (please specify): ________________________________________________

Why did you choose this drug (other than Truvada® or generic Truvada®)? (*2 answers maximum*)

- You trust the effectiveness of (all) HIV treatments
- You use Truvada® in general and supplement from time to time with other medication
- You only have access to other medication
- Other (please explain): ________________________________________________

What year did you take PrEP for the first time? *(for example: 2012)*

________________________________________________________________

When you took PrEP for the first time, it was:

- After you made some inquiries yourself
- After a doctor or a health care worker told you about it
- After a friend and/or a partner told you about it
- After you heard about it from a NGO/charity
- Other (please specify) ________________________________________________

Have you ever skipped a dose or stopped temporarily?

- Yes
- No

**In the past 3 months**, how many times and for how long did you take breaks?
 *(For example, if you stopped once for 4 days and twice for 6 days, click in the row "4 days" and "6 days" and enter "once" with "4 days" and "2-4 times"  under "6 days")*

|  | Never | Once | 2-4 times | 5-7 times | More than 8 times |
| --- | --- | --- | --- | --- | --- |
| 1 day |  |  |  |  |  |
| 2 days |  |  |  |  |  |
| 3 days |  |  |  |  |  |
| 4 days |  |  |  |  |  |
| 5 days |  |  |  |  |  |
| 6 days |  |  |  |  |  |
| 7 days |  |  |  |  |  |
| >7 days |  |  |  |  |  |

*Think about the last break in your PrEP*. What were the main reasons? *(3 answers maximum)*

- I forgot to take my tablets
- I ran out of tablets
- I was no longer having sex
- I changed partners
- I experienced side effects
- I was no longer with my main partner
- I preferred other prevention tools (condoms, regular testing, choosing partners with an undetectable viral load, etc.)
- I could not afford to get PrEP anymore
- I could not obtain PrEP anymore
- Other (please explain): ________________________________________________

How often do you take PrEP?

- Several times a week
- Once or twice a week
- Less than once a week
- Once a month
- Less than once a month

You take PrEP:

- Every time you have sex
- Just when you think it's risky

In general, how have you obtained your PrEP tablets? *(more than one answer possible)*

- With a medical prescription from a doctor (paying myself)
- With a medical prescription for curative use (you said that you were HIV-positive)
- By using HIV treatments prescribed as post-exposure prophylaxis (PEP) (you said that you were HIV-negative and you had a risky behaviour)
- From a NGO/charity
- An HIV-positive friend gives/gave you their HIV treatment
- From a participant in a PrEP clinical trial
- From another PrEP user (outside of a clinical trial)
- By purchasing them online
- By purchasing generics in other countries of the world
- By buying them in the street, at leisure-time/entertainment or sexual venues
- Other (please specify) ________________________________________________

On average, on a monthly basis, how much do/did you spend on PrEP?

________________________________________________________________

In which currency did you enter the amount?

- Euro
- CHF
- Danish Crone
- Romanian Leu
- GBP
- Other (please specify) ________________________________________________

What percentage of your monthly income does this amount represent?

- 5-10%
- 10-25%
- 25-50%
- >50%

Have you told those around you that you are taking PrEP?

|  | Yes | No | Not applicable |
| --- | --- | --- | --- |
| Friends |  |  |  |
| HIV-positive friends |  |  |  |
| Family |  |  |  |
| Main sex partner |  |  |  |
| Occasional sex partners |  |  |  |
| Doctors or other medical personnel |  |  |  |
| Members of a NGO/charity |  |  |  |
| I publicly display it on dating sites/apps |  |  |  |
| I advocate for PrEP (I tell everyone around me that I'm using it) |  |  |  |

Have you had any medical check-ups specifically related to your taking PrEP (for example, tests for your kidneys)?

- Yes
- No

How often do/did you get medical check-ups with regard to PrEP?

- Only once, before I started
- Every 3 months
- Irregularly

When you are on PrEP, do you accept that your partners want to use a condom?

- Never
- Rarely
- From time to time
- Nearly always
- Always

**Since you started taking PrEP**, would you say that...

|  | A lot lower than before | Lower than before | The same | Higher than before | A lot higher than before |
| --- | --- | --- | --- | --- | --- |
| Your condom use is: |  |  |  |  |  |
| Your frequency of HIV testing: |  |  |  |  |  |
| Your risk of infection from HIV: |  |  |  |  |  |

**Since you started taking PrEP**, would you say that:

|  | A lot worse | Worse | The same | Better | A lot better |
| --- | --- | --- | --- | --- | --- |
| Your quality of **life in general** is: |  |  |  |  |  |
| The quality of your **sex life** is: |  |  |  |  |  |

**This is the end of the questionnaire. Thank you very much for taking part in the survey. If you have any comments, feedback or wish to add any additional information, please fill in the box below.**

________________________________________________________________

________________________________________________________________

________________________________________________________________

________________________________________________________________

________________________________________________________________
